# Supplementary material for: Anterior fontanelle size among term neonates on the first day of life born at University of Gondar Hospital, Northwest Ethiopia
Source: PLoS One. 2018 Oct 26;13(10):e0202454. doi: 10.1371/journal.pone.0202454 (PMC6203250; doi:10.1371/journal.pone.0202454)
Supplement: S1 File — (PDF) [file pone.0202454.s001.pdf]

Supporting information

S 1. Confidentiality and informed consent statement

Introduction

A study on AF size among term neonates on the first day of life born at UoGH, Northwest Ethiopia, 2018

ID Number ____________

Date of Interview _______

Time at the beginning of the interview_____

Hello! Madam/Sir my name is __________________

I am Human Anatomy post-graduate student at University of Gondar and I have been working for University of Gondar and Hospital since 2016. Now, we are working on research that tries to address AF size related problem and its related variables in this teaching hospital. This improves the clinical evaluation and gives useful information that can be used to follow the developmental status of the neonates and infants on the community. You or your term neonate has been chosen randomly for the study.

Confidentiality and informed consent statement

I am going to ask you questions about AF size related variables before assessing your neonate. Your name will not appear on this checklist and all the information you provide to me will be strictly confidential. You are not obliged to answer any questions that you don't wish to answer, and you can put an end to this interview at any time, if you wish to do so. Your participation in this study does not involve any direct risk or benefit for you or your neonate. However, it is very useful since your answers, as well as those of other participants, will help to get average AF value of Gondar neonates and gives useful information for clinicians in order to care the neonates and infants in the community.

Would you like to participate in the study?

1. Yes
2. No
