# Supplementary material for: Anterior fontanelle size among term neonates on the first day of life born at University of Gondar Hospital, Northwest Ethiopia
Source: PLoS One. 2018 Oct 26;13(10):e0202454. doi: 10.1371/journal.pone.0202454 (PMC6203250; doi:10.1371/journal.pone.0202454)
Supplement: S2 File — (PDF) [file pone.0202454.s002.pdf]

Supporting information

S 2. Consent form and ethical clearance

(For those able to write and read)

I have read and understood well the condition stated above and I can withdraw from the study at any time and I understand that there is no risk on being participate and no incentive to be given when I participate in the study. Therefore, I am willing to participate in the study.

Signature___________________ Date____________ 2010 E.C

Thank you!!!

Result Code _____ (1= Completed, 2= partially completed, 3= refused, 4=other)

Time at the end of the interview __________

Investigator name & Signature __________________

ጎንደር ዩኒቨርሲቲ
የሕክምና ት/ቤት
ጎንደር፣ ኢትዮጵያ

University of Gondar
School of Medicine
Gondar, Ethiopia

College of Medicine & Health Sciences

Ref. [illegible] /2007

Date: [illegible] 2017

# TO WHOM IT MAY CONCERN

Subject: - Ethical Clearance

Mr. Mohammed Oumer is a Post Graduate Students department of Human Anatomy, School of Medicine, College of Medicine and Health Sciences, University of Gondar. He is planning to conduct research entitled with **"Anterior fontanel Size in term neonates on the first day of life born at University of Gondar Hospital, Northwest Ethiopia."** His proposal has been submitted to the School of Medicine for Ethical Review Board and Approved as Ethically sound research.

The very kind co- operation of your organization is of great importance for the success of the thesis work;

The School of Medicine appreciates your cooperation in advance.

With regards!

[illegible]

Dr. Abebe Muche
Assistant Professor of Anatomy
Chair, Ethical review board

ጎንደር ዩኒቨርሲቲ University of Gondar
የሕክምና ት/ቤት School of Medicine
College of Medicine & Health Sciences

[illegible]

25/03/10

Menyichil Tamer Tizazu

የፖ.ሣ.ቁ
P.O. Box 196

ጎንደር ኢትዮጵያ
Gondar, Ethiopia

ቴሌግራም ጤ.ኮ
Cable A.A.U. PH.
Fax -251-058-114 1240
251-058 114 1233
251-058 114 1235
URL.Address:- www.ugondar.edu.et

ስልክ

Telephone PBX	058 111 0174
President's Office	058 114 1231
V/P/ for Academic & Research	058 114 1236
V/President for Admin.	058 114 1238
Human Resource	058 111 0157
School of Pharmacy	0581115679
School of Medicine	0581115584

መልስ ሲጽፉልን የእኛን ቁጥር ይጥቀሱ፡፡
In Replying, please Quote our Ref. No A.S/አ.ሰ
