# Supplementary material for: Anterior fontanelle size among term neonates on the first day of life born at University of Gondar Hospital, Northwest Ethiopia
Source: PLoS One. 2018 Oct 26;13(10):e0202454. doi: 10.1371/journal.pone.0202454 (PMC6203250; doi:10.1371/journal.pone.0202454)
Supplement: S3 File — (PDF) [file pone.0202454.s003.pdf]

Supporting information

S 3: English data collection checklist

Data collection checklist

Name of the institution ___________________________

Part One: Interview with the Mother

S.No.	Questions	Choice of responses	Code
1.	Identification Number		
2.	Age of the Mother	…………. in years	
3.	Address (place of residence); Kebele ___<br>Woreda __ Zone __ Other, specify____	1. Urban<br>2. Rural	
4.	Marital status	1.Married<br>2.Widowed<br>3. Divorced<br>4. Single<br>5. Other, specify	

5.	Educational status	1. Unable to read and write<br>2. Read only<br>3. Only able to read and write<br>4. From 6-12 grades<br>5. Certificate holder<br>6. Diploma holder<br>7. Degree holder and above	
6.	Occupation	1.House wife<br>2.Merchant<br>3.Goverment employee<br>4.Daily laborer<br>5. Farmer<br>6. Student<br>7.Other , specify	
7.	Monthly income (In Ethiopian Birr)	1. ≤ 600<br>2. 601-1650<br>3.1651- 3200<br>4. 3201-5250<br>5. ≥ 5251<br>6. No income	

Part Two: Pregnancy

S.No.	Questions	Choice of responses	Code
1.	Parity/ Birth order		
2.	Gestational age at birth	…………..weeks	

Part Three: Labour

S.No.	Questions	Choice of responses	Code
1.	Onset of labour	1.Spontaneous<br>2.Induced	
2.	Duration of labour	……….. hours	
3.	Mode of delivery	1. Spontaneous vertex delivery<br>2. Caesarean section	

Part Four: Outcome

S. No.	Questions	Choice of responses	Code
1.	Birth weight	……………… gm	
2.	Gender of neonate	1. Male<br>2. Female	

Part Five: Newborn

S.No.	Questions	Choice of responses	Code
1.	Age in hours	……………… hrs	
2.	AF length	……………….cm	
3.	AF width	……………….cm	
4.	Head circumference	……………….cm	
5.	Does your neonate have any medical problem (or attending physician)?	1.Yes<br>2. No<br>3. If yes, specify	

1. Date of birth __________________________________
2. Form completed on __________________________________
3. About AF

First mark of AF

____

Second mark of AF

____
