# Supplementary material for: Anterior fontanelle size among term neonates on the first day of life born at University of Gondar Hospital, Northwest Ethiopia
Source: PLoS One. 2018 Oct 26;13(10):e0202454. doi: 10.1371/journal.pone.0202454 (PMC6203250; doi:10.1371/journal.pone.0202454)
Supplement: S4 File — (PDF) [file pone.0202454.s004.pdf]

Supporting information

S 4. Amharic Version checklist

የጎንደር ዩንቨርስቲ

የህክምና እና ጤና ሣይንስ ኮሌጅ

የህክምና ትምህርት ቤት አናቶሚ ትምህርት ክፍል

ስለፊተኛው እርግብግቢት መጠን ለማጥናት የሚሞላ ቸክሊስት።

መግቢያ

የጨቅላ ህፃናት የፊተኛው እርግብግቢት መጠን ልኬት በ ጎንደር ዩንበርስቲ ሆስፒታል በተወለዱ የመጀመሪያ ቀን የሚሰራ ጥናት።

የመለያ ቁጥር -----------

ቃለ መጠይቅ የተደረገበት ቀን -----

የተጀመረበት ግዜ -------------

እሜቴ/ አባቴ ስሜ -------------- እባላለሁ፡፡

የምሰራው በጎንደር ዩንቨርስቲ ቅጥር ግቢ ውስጥ ሲሆን አሁን የአናቶሚ ትምህርት ክፍል የሁለተኛ ዲግሪ ተመራቂ ተማሪ ነኝ፡፡ በፊተኛው እርግብግቢት የመጠን ጥናት እየሰራን እንገኛለን ስለዚህ የሚሰበሰበው መረጃ ለማህበረሰባችን ከፍተኛ አስተዋጥኦ እንደሚያበረክት ለንገልጥልዎ እንወዳለን ፡፡

ሚስጥርን ስለመጠበቅ

ልጅዎ ለዚህ ጥናት በዕጣ ስለተመረጠ ስለ ፊተኛው እርግብግቢትም ሆነ ስለ እርስዎ አንዳንድ ጥያቄ እንጠይቀዎት አለን፡፡

ስመዎት በመጠይቁ ላይ የማይጠቀስ ሲሆን የምትሰጡን መረጃ ሚስጥር የተጠበቀ ነው፡፡ በጥናቱ መሳተፍሽ ባንችም ሆነ በልጁ/ጇ ምንም አይነት ጉዳት የማያደርስ ሲሆን ያለመሳተፍም መብት እንዳለሽ ልነግርሽ እወዳለሁ፡፡

በጥናቱ ላይ መሳተፍ ይፈልጋሉ !

የስምምነት ፍቃድ (ማንበብ እና መጻፍ ለሚችሉ)

ከላይ የተጻፈውን በደንብ አንብቤ ተረድቸው አለሁ። በጥናቱ ላይ መሳተፍ በኔም ሆነ በልጄ ላይ ምንም አይነት ችግር እንደማይደርስ ፤ ጥቅማጥቅምም እንደሌለው እንዲሁም በማነኛውም ሰአት ካልተመቸኝ የማቋረጥ መብት እንዳለኝ ተነግሮኛል።

በመሆኑም በጥናቱ ላይ ለመሳተፍ ዝግጁ ነኝ ።

ፊርማ --------------------- ቀን --------------------------------2010

አመሰግናለሁ።

የዉጤቱ ኮድ፡ 1. ሙሉ በሙሉ የተሞላ   2. በከፊል የተሞላ   3. የተቋረጠ   4. ሌላ

ፎርሙን የሞላው ስምና ፊርማ ---------------------------

እናመሰግናለን !!!

የመረጃ መሰብሰቢያ ቅጽ (ቸክሊሰት)

የመስሪያቤቱ ስም -----------------------------

ክፍል 1፡ ስለ ህጻኑ እናት የተዘጋጀ መጠይቅ

ተ.ቁ	ጥያቄዎች	አማራጮች	ኮድ
1.	መለያ ቁጥር		
2.	የእናት እድሜ በአመት	-------------- በ አመት	
3.	አድራሻ	1. ከተማ<br>2. ገጠር	
4.	የጋብቻ ሁኔታ	1. ያገባች<br>2. ባሏ የሞተባት<br>3. የተፋታች<br>4. ያላገባች<br>5. ሌላ ካለ ጥቀሽ	
5.	የትምህርት ሁኔታ	1. ማንበብ እና መጻፍ የማትችል<br>2. ማንበብ ብቻ የምትችል<br>3. ማንበብ እና መጻፍ ብቻ የምትችል<br>4. ከ 6-12 ክፍል የተማረች<br>5. ሰርትፍኬት ያላት<br>6. ዲፕሎማ ያላት<br>7. ዲግሪ እና ከዛ በላይ ያላት	
6.	የእናት ስራ	1. የቤት እመቤት<br>2. ነጋዴ<br>3. የመንግስት ሰራተኛ<br>4. የቀን ሰራተኛ<br>5. ነጋዴ<br>6. ተማሪ<br>7. ሌላ ካለ ጥቀሽ	

7.	የወር ገቢ (በኢትዮጵያ ብር)	1. ≤ 600<br><br>2. 601-1650<br><br>3.1651- 3200<br><br>4. 3201-5250<br><br>5. ≥ 5251<br><br>6. ገቢ የለባት	

ክፍል 2: ስለ እርግዝና

ተ.ቁ	ጥያቄዎች	አማራጮች	ኮድ
1.	ስንተኛ ልጅሽ ነዉ/ነች		
2.	የእርግዝና እድሜ በሳምንት	------------ሳምንት	

ክፍል 3: ስለ ምጥ

ተ.ቁ	ጥያቄዎች	አማራጮች	ኮድ
1.	የምጡ አጀማመር	1. በራሱ<br>2. በምጥ መርፌ	
2.	የምጡ ርዝማኔ በሰአት	---------በሳአት	
3.	የወሊድ አይነት	1. በማህጸን አምጣ<br>2. በቀዶ ጥገና	

ክፍል 4: አውትካም

ተ.ቁ	ጥያቄዎች	አማራጮች	ኮድ
1.	የልጅ ክብደት በ ግራም	------------ግራም	
2.	የህጻኑ ጾታ	1. ወንድ<br>2. ሴት	

ክፍል 5: ስለ ልጁ

ተ.ቁ	ጥያቄዎች	አማራጮች	ኮድ
1.	የልጁ ዕድሜ በሰዓት	-------------------በ ሰዓት	
2.	የፊተኛው እርግብግቢት ቁመት በ ሴ.ሜ	------------------ሴሜ	
3.	የፊተኛው እርግብግቢት ስፋት በ ሴ.ሜ	-------------------ሴሜ	
4.	የጭንቅላት ዙር በ ሴ.ሜ	-------------------ሴሜ	
5.	ልጁ የጤና እንከን አለበት (ወይም ለሃኪሙ)?	1. አለ<br>2. የለም<br>3. ካለ ምን አይነት	

1. የተወለደበት ቀን ________________________________

2. ቸክሊስት የተሞላበት ቀን ________________________________

3. ስለ አንተሪር ፎንታንል

አንደኛ ማርክ

____

ሁለተኛ ማረክ

____
